# *Qiliqiangxin* Enhances Cardiac Glucose Metabolism and Improves Diastolic Function in Spontaneously Hypertensive Rats

**DOI:** 10.1155/2017/3197320

**Published:** 2017-06-19

**Authors:** Jingfeng Wang, Zhiming Li, Yanyan Wang, Jingjing Zhang, Weipeng Zhao, Mingqiang Fu, Xueting Han, Jingmin Zhou, Junbo Ge

**Affiliations:** ^1^Department of Cardiology, Shanghai Institute of Cardiovascular Diseases, Zhongshan Hospital, Fudan University, Shanghai, China; ^2^Department of Cardiology, People's Hospital of Nanbu County, Nanchong, Sichuan, China; ^3^Department of Cardiology, Shandong University, Jinan, Shandong, China

## Abstract

Cardiac diastolic dysfunction has emerged as a growing type of heart failure. The present study aims to explore whether* Qiliqiangxin *(QL) can benefit cardiac diastolic function in spontaneously hypertensive rat (SHR) through enhancement of cardiac glucose metabolism. Fifteen 12-month-old male SHRs were randomly divided into QL-treated, olmesartan-treated, and saline-treated groups. Age-matched WKY rats served as normal controls. Echocardiography and histological analysis were performed. Myocardial glucose uptake was determined by ^18^F-FDG using small-animal PET imaging. Expressions of several crucial proteins and key enzymes related to glucose metabolism were also evaluated. As a result, QL improved cardiac diastolic function in SHRs, as evidenced by increased *E*′/*A*′and decreased *E*/*E*′ (*P* < 0.01). Meanwhile, QL alleviated myocardial hypertrophy, collagen deposits, and apoptosis (*P* < 0.01). An even higher myocardial glucose uptake was illustrated in QL-treated SHR group (*P* < 0.01). Moreover, an increased CS activity and ATP production was observed in QL-treated SHRs (*P* < 0.05). QL enhanced cardiac glucose utilization and oxidative phosphorylation in SHRs by upregulating AMPK/PGC-1*α* axis, promoting GLUT-4 expression, and regulating key enzymes related to glucose aerobic oxidation such as HK2, PDK4, and CS (*P* < 0.01). Our data suggests that QL improves cardiac diastolic function in SHRs, which may be associated with enhancement of myocardial glucose metabolism.

## 1. Introduction

Heart failure with preserved ejection fraction (HFpEF) serves as a growing type of cardiac dysfunction, the morbidity of which accounts for up to 50% of all heart failure patients and has been gradually rising over the past 2 decades [1]. Multiple mechanisms are likely involved in HFpEF, but no single abnormality could explain its pathophysiology. The abnormality most commonly seen in patients with HFpEF is impaired diastolic function, which is manifested by increased left ventricular (LV) stiffness, impaired LV relaxation, and contractile reserve [2]. Myocardial hypertrophy and interstitial fibrosis contribute to high LV stiffness and heart failure development. However, there still exist some other elements participating in the progress of HFpEF.

Recent studies have demonstrated that LV diastolic dysfunction is associated with impaired mitochondrial oxidative phosphorylation, reduced glucose oxidation, and adenosine triphosphate (ATP) levels [3–5]. Insulin resistance and glucose metabolism disorders could also be observed in patients with diastolic dysfunction [6, 7]. In addition, Hermida et al. reported that statin treatment reverses myocardial remodeling and improves ventricular relaxation through AMP-activated protein kinase (AMPK)-mediated antifibrotic effects in a mouse model of metabolic syndrome [8]. Li et al. revealed that trimetazidine significantly improved systolic and diastolic function in hearts of* db/db* mice, in which activation of AMPK and decreased expression of peroxisome proliferator-activated receptor coactivator-1*α* (PGC-1*α*) were involved [9]. All these data suggest that myocardial glucose metabolism plays an important role in cardiac diastolic function.

*Qiliqiangxin *(QL), a traditional Chinese medicine extracted from 11 distinct herbs including* Radix Astragali*,* Aconite Root*,* Ginseng*,* Salvia miltiorrhiza*,* Semen Lepidii Apetali*,* Cortex Periplocae Sepii Radicis*,* Rhizoma Alismatis*,* Carthamus tinctorius*,* Polygonatum Odorati*,* Seasoned Orange Peel*, and* Rumulus Ginnamomi*, has proved to be effective and safe in the management of chronic heart failure due to either ischemic injury or pressure overload [10–12]. Previous studies have documented that QL could improve cardiac energy metabolism and mitochondrial function in pressure overload heart failure rats by activating AMPK/PGC-1*α* axis [13]. It could also enhance metabolism in cardiomyocytes, which is linked to increased mitochondrial content and mitochondrial biogenesis via activation of PGC-1*α* [14]. However, it remains unclear whether QL regulates myocardial glucose metabolism in spontaneously hypertensive rats (SHRs). Long-standing hypertension triggers concentric hypertrophy and increases LV passive stiffness, which leads to impaired LV diastolic function and then to the development of decompensated heart failure. It replicates the classical progression of clinical hypertensive heart disease [15–17]. The present study aims to explore whether chronic administration of QL could enhance myocardial glucose metabolism and prevent LV diastolic dysfunction and cardiac remodeling in an aging SHR model.

## 2. Materials and Methods

### 2.1. Animal Model and Vegetal Material

Fifteen 12-month-old male SHRs and five age-matched male Wistar Kyoto (WKY) rats were purchased from Shanghai Institute of Hypertension. Animals were housed in a temperature- and humidity-controlled facility with a 12 : 12 h light-dark cycle and fed with a standard diet and water ad libitum. QL compounds were provided by Yiling Pharmaceutical Corporation (Shijiazhuang, China). The origin, harvest time, medicinal composites, and processing technology of the 11 pharmaceutical ingredients of QL were strictly authenticated and standardized on the basis of marker compounds to achieve quality control according to the Chinese Pharmacopoeia 2005 (National Pharmacopoeia Committee, 2005) [13]. Drug powder suspension was prepared at a concentration of 0.2 g/mL each day before administration.

### 2.2. Study Protocol and Drug Administration

All experimental protocols were performed in compliance with the Guide for the Care and Use of Laboratory Animals published by the US National Institutes of Health (Publication number 85-23, revised 1996) and approved by the Animal Care Committee in Zhongshan Hospital, Fudan University. SHRs were randomly assigned into three treatment groups (*n* = 5 for each): SHR + QL group, fed with QL powder suspension (0.5 g/kg·d); SHR + olmesartan group, fed with olmesartan (2.5 mg/kg·d); SHR + saline group: given equal volume of saline alone. WKY rats administrated with same volume of saline were considered as normal controls. All treatment was carried out by oral gavage once daily continuously for two months.

Before drug administration, systolic blood pressure and heart rate (HR) were measured by tail-cuff plethysmography (BP-2000, Visitech Systems Inc., USA) and body weight was determined by an electronic scale. Both LV systolic and diastolic function were evaluated by transthoracic high-frequency echocardiographic studies. After drug intervention, animals were subjected to systolic blood pressure measurement and transthoracic echocardiography again, together with small-animal positron emission tomography (PET) imaging, followed by euthanasia.

### 2.3. Echocardiography

Before echocardiography, all rats were anesthetized with an intraperitoneal injection of ketamine hydrochloride (50 mg/kg). After animals were transferred to a heated platform, the precordial region was shaved and a warm acoustic coupling gel was gently applied to ensure optimal image quality. Echocardiography was performed in a supine position with a heart rate around 200–300 beats/minute using a high-frequency 17.5-MHz linear transducer connected to a high-resolution ultrasound system (Vevo770 High Resolution Imaging System, Visual Sonics, Toronto, Canada). M-mode and 2-dimensional echocardiographic images were obtained from parasternal long- and short-axis views for assessment of LV end-diastolic diameter (LVEDD), LV end-systolic diameter (LVESD), LV posterior wall thickness (LVPW), interventricular septum thickness in diastole (IVS), LV ejection fraction (LVEF), and fractional shortening (LVFS). The early peak transmitral flow velocity (*E*) to late peak transmitral flow velocity (*A*) ratio (*E*/*A*) and the deceleration time of *E* wave (DT) were measured using pulsed-wave Doppler imaging from apical 4-chamber view. Meanwhile, tissue Doppler imaging (TDI) was performed at the level of lateral mitral annulus to record peak early (*E*′) to late (*A*′) diastolic tissue velocity ratio (*E*′/*A*′). Ratio of *E*/*E*′ was also calculated. All measurements were completed by one experienced echocardiographer and averaged for at least 4 consecutive cardiac cycles.

### 2.4. Small-Animal PET Imaging

Animals were deprived of food but allowed water ad libitum for 6 hours before the study. Under anaesthesia with 50 mg/kg ketamine hydrochloride, rats received an intravenous injection of 2-deoxy-2-[18F]fluoro-D-glucose (^18^F-FDG) (approximately 1250 *μ*Ci/kg) via the tail vein. PET imaging acquisition was performed 2 h after ^18^F-FDG administration and continued for 30 min. All in vivo imaging was obtained using a small-animal PET system (Metis PET, Madic, Shandong, China). Emission scan was acquired in one bed position with a transverse field of view of 80 mm and an axial extent of 59.6 mm. The spatial resolutions in the transverse and axial directions were less than 1.3 mm. PET images were reconstructed using the 3-dimensional ordered-subsets expectation-maximization algorithm (eight iterations and six subsets) without electrocardiographic gating. Data were reconstructed into 90 × 90 × 60 matrices with a corresponding voxel size of 0.9 × 0.9 × 0.9105 mm and a thickness of 0.9 mm. No corrections were made for attenuation or scatter. Volumetric sampling was applied to delineate 3D tracer distributions of ^18^F-FDG uptake throughout the LV myocardium. Image analysis was carried out using Metis Viewer Version 1.0 for Windows. Standardized uptake value (SUV) was applied to evaluate ^18^F-FDG uptake by the heart, which was calculated and normalized according to body weight and the injected dose of ^18^F-FDG: SUV = radioactivity in the heart by PET (Bq/mL)/injection dosage of ^18^F-FDG (Bq) per body weight (g).

### 2.5. Plasma Biomarkers Assay and Histological Analysis

All rats were euthanized one day after PET imaging was completed. Blood samples were withdrawn from inferior vena cava. Plasma was separated through centrifugation and concentrations of N-terminal probrain natriuretic peptide (NT-proBNP), tumor necrosis factor-*α* (TNF-*α*), and transforming growth factor-*β*1 (TGF-*β*1) were detected using enzyme linked immunosorbent assay (ELISA) kit (Cloud-Clone Corp, USA) according to the manufacturer's instructions. After euthanasia, hearts were rapidly excised with the LV weighed to calculate LV mass index (LVMI), expressed as ratio of LV weight (mg) to total body weight (g). After being rinsed several times, transversely dissected heart tissues were fixed with 4% paraformaldehyde, embedded in paraffin, and cut into 5 *μ*m thick sections for hematoxylin-eosin (HE) staining, Masson trichrome staining, and TUNEL staining. Remaining tissue granules were kept at −80°C for further analysis. Cardiomyocyte cross-sectional area (CSA) was then assessed under 400x high-powered field of HE staining. The extent of fibrosis was evaluated by dividing the fibrotic area by total tissue area of LV under 200x high-powered field of Masson trichrome staining. TUNEL staining was performed using an in situ cell death detection Kit (Roche, Germany) according to the manufacturer's instructions. The ratio of TUNEL-positive cells to total cardiomyocytes was calculated under 400x high-powered field. Five randomly selected fields were examined and averaged for each specimen. Photographs were taken and analyzed by a high-resolution digital image analysis system (Qwin V3, Leica, Germany).

### 2.6. Western Blot

Approximately 30 mg heart tissues collected from LV were homogenized in lysis buffer (Beyotime, Nantong, China) supplemented with a cocktail of protease and phosphatase inhibitors (Sigma Chemical Co., St Louis, MO) and centrifuged. Protein lysates from each sample were then loaded onto 12% SDS-polyacrylamide gels (40 *μ*g/well). Following electrophoresis, separated proteins were transferred to polyvinylidene difluoride (PVDF) membrane (Thermo Scientific, USA). The blotted membrane was then incubated with primary antibodies specific for AMPK*α* and phospho-AMPK*α*^Thr172^ (Cell Signaling Technology, Inc., USA), glucose transporter-1 (GLUT-1) (Cell Signaling Technology, Inc., USA), glucose transporter-4 (GLUT-4) (Cell Signaling Technology, Inc., USA), PGC-1*α* (Abcam, UK), and *β*-actin (Kangcheng Biological Co., China) overnight at 4°C. After washes, blots were then incubated with horseradish peroxidase-conjugated secondary antibody (Abbkine, Inc., USA) for 2 h. Finally, immunocomplexes were visualized by ECL Plus (Thermo Scientific, USA) and analyzed with Image Lab Software (Bio-Rad Laboratories, Inc., USA). Quantification of relative protein expression was normalized to *β*-actin and expressed as fold change.

### 2.7. Measurement of Myocardial ATP Content and Citrate Synthase (CS) Activity

Myocardial ATP content was detected using an ATP Assay Kit (Beyotime, China) according to the manufacturer's instructions. Approximately 20 mg LV myocardial tissues were lysed and homogenized, followed by centrifugation at 12000*g* for 5 minutes. Supernatant was mixed with diluted luciferase reagent. The emitted light was measured by a microplate luminometer (Thermo Scientific, USA) and ATP content was calculated from a standard curve. Finally ATP level was normalized to protein concentration, which was determined with a BCA protein assay kit (Beyotime, China). CS activity was measured using an Citrate Synthase Activity Assay Kit (Sigma-Aldrich, USA). Briefly, 10 mg tissue samples were homogenized in CS assay buffer and centrifuged at 10000*g* for 5 minutes. Supernatants were diluted appropriately and mixed with reaction mixes. Both the initial and final absorbance at 412 nm were measured for each sample for calculating the changes in absorbance (Δ*A*_412_). Sample blank Δ*A*_412_ value was subtracted from the sample Δ*A*_412_ reading to obtain the corrected measurement. Sample values were then read off the standard curve and expressed as nmole per minute per milligram of total protein.

### 2.8. Quantitative Real-Time Polymerase Chain Reaction (PCR)

Several key enzymes involved in glycolysis and aerobic oxidation were detected in our study. Total RNA was isolated from the LV tissue samples (approximately 100 mg) using TRizol reagent according to the manufacturer's protocol (Invitrogen, USA). cDNA synthesis was performed in a 20 *μ*L reaction volume using PrimeScript™ RT reagent kit (Takara, Japan). Quantitative real-time PCR was performed on an Applied Biosystems® 7500 Real-Time PCR Detection System (Life Technologies, USA) using SYBR® Premix Ex Taq™ kit (Takara, Japan). PCR thermal cycling involved a denaturing step at 95°C for 30 s followed by 40 cycles of annealing step at 95°C for 5 s and extension at 60°C for 34 s with each sample analyzed in triplicate. Sequences for specific primers were as follows: hexokinase-2 (HK2): sense, 5′-GACTTCGCTCCACCATCG-3′ and antisense, 5′-GCCTCCTCACTGCCTTATG-3′; pyruvate dehydrogenase kinase-4 (PDK4): sense, 5′-GCGATGTGGTAGCAGTAGTC-3′ and antisense, 5′-ATGTGGTGAAGGTGTGAAGG-3′; CS: sense, 5′-GGAACACACTCAACTCGGGA-3′ and antisense, 5′-ACCCCACTGTGAGCATCTACG-3′; *β*-actin: sense, 5′-ACCACAGTCCATGCCATCAC-3′ and antisense, 5′-TCCACCACCCTGTTGCTGTA-3′. Quantification of relative mRNA expression was normalized to *β*-actin as endogenous control by the standard 2^−ΔΔCt^ method.

### 2.9. Statistical Analysis

Values were expressed as mean ± standard deviation (SD). Data between different groups were compared using one-way ANOVA. *P* < 0.05 was considered statistically significant. All statistical analyses were carried out with SPSS software (version 19.0, SPSS Inc., USA).

## 3. Results

### 3.1. QL Improved Cardiac Diastolic Function Independent of Blood Pressure

**Figure 1:**
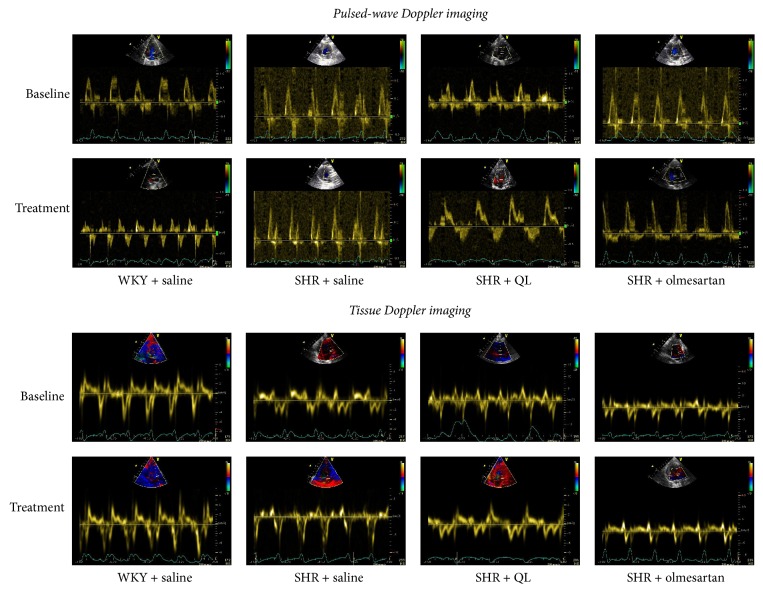
Left ventricular diastolic function assessed by transmitral pulsed-wave Doppler imaging and tissue Doppler imaging at the level of lateral mitral annulus from apical 4-chamber view. Maximal flow velocities of the early (*E*)/late (*A*) waves and maximal tissue velocity of early (*E*′)/late (*A*′) diastolic waves were recorded correspondingly. QL improved diastolic function in SHR, as illustrated by increased *E*′/*A*′ ratio instead of *E*/*A* ratio.

As is shown in Table 1, a significant decline of LV diastolic function could be observed in SHRs in comparison with the age-matched WKY controls, as was characterized by decreased *E*′/*A*′ ratio and increased *E*/*E*′ ratio and DT. QL ameliorated LV diastolic function in SHRs with increased *E*′/*A*′, decreased *E*/*E*′, and DT when compared with SHR + saline group (Figure 1). QL also attenuated LV hypertrophy demonstrated by decreased IVS and LVPW in SHRs. Furthermore, SHRs demonstrated a significantly higher LVEF and LVFS than WKY controls, whereas no difference was observed concerning LV systolic function after intervention among the three SHR groups, since it is confirmed that systolic dysfunction appeared at 18 months of age in SHR [18]. Olmesartan showed a similar beneficial effect on cardiac diastolic function in SHRs. Interestingly, QL had no impact on systolic blood pressure though olmesartan therapy significantly lowered blood pressure compared with SHR + saline group, indicating that QL improved cardiac diastolic function in SHRs independent of blood pressure (Table 1).

### 3.2. QL Decreased Plasma Levels of NT-ProBNP, TNF-*α*, and TGF-*β*1, Reduced LVMI, and Alleviated LV Hypertrophy, Fibrosis, and Apoptosis

**Figure 2:**
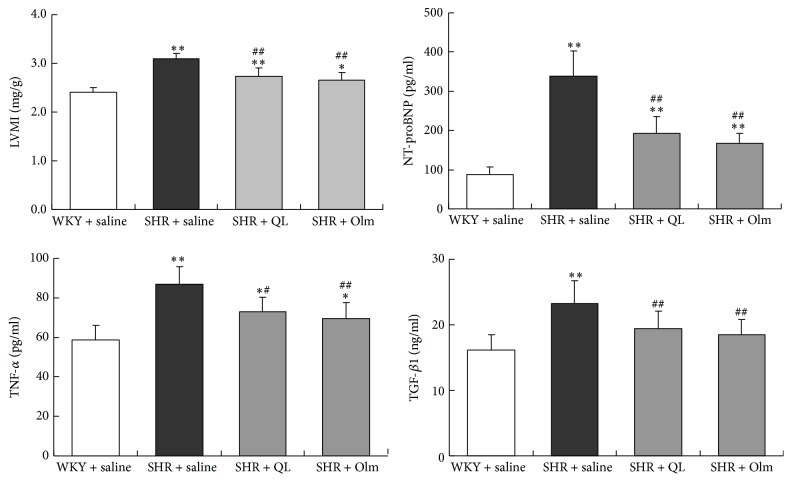
Left ventricular mass index (LVMI) and plasma levels of NT-proBNP, TNF-*α*, and TGF-*β*1 shown in all the four groups. Olm, olmesartan; ^*∗*^*P* < 0.05* versus* WKY + saline group; ^*∗∗*^*P* < 0.01* versus* WKY + saline group; ^#^*P* < 0.05* versus* SHR + saline group; ^##^*P* < 0.01* versus* SHR + saline group.

**Figure 3:**
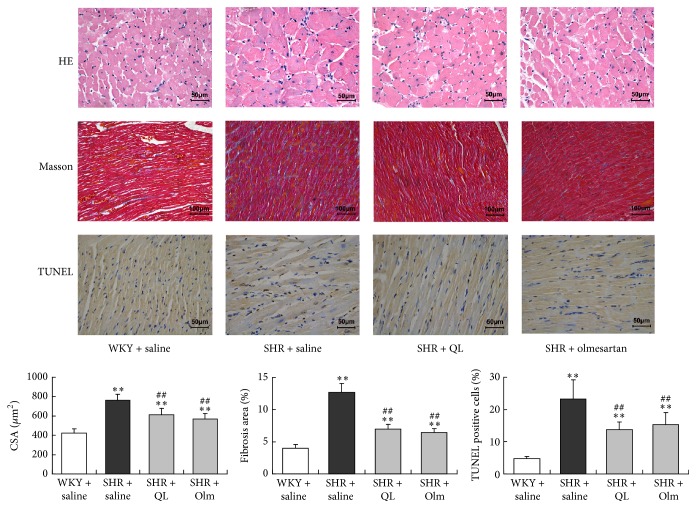
Photography of HE staining (400x), Masson's trichrome staining (200x), and TUNEL staining (400x) for assessment of cardiomyocyte cross-sectional area (CSA), percentage of fibrosis area, and cardiomyocyte apoptosis, respectively. Olm, olmesartan; ^*∗∗*^*P* < 0.01* versus* WKY + saline group; ^##^*P* < 0.01* versus* SHR + saline group.

In our study, SHRs demonstrated markedly higher plasma levels of NT-proBNP, TNF-*α*, and TGF-*β*1 than age-matched WKY rats. QL and olmesartan significantly decreased these plasma biomarkers (*P* < 0.05). Compared with age-matched WKY rats, a significantly increased LVMI was observed in SHR + saline group, which was also significantly reduced in QL and olmesartan group (*P* < 0.01) (Figure 2). Histological studies demonstrated that myocardial samples from the SHR + saline group developed obvious cardiac hypertrophy, fibrosis, and myocyte apoptosis. QL and olmesartan administration significantly alleviated LV hypertrophy, fibrosis, and apoptosis, as exhibited by decreased cardiomyocyte CSA, reduced percentage of fibrotic deposits, and fewer TUNEL-positive cells in the LV sections (Figure 3). These findings suggested that QL reversed myocardial remodeling in SHR, which contributed to improve LV diastolic filling properties.

### 3.3. QL Enhanced Myocardial Glucose Uptake Illustrated by PET Imaging

**Figure 4:**
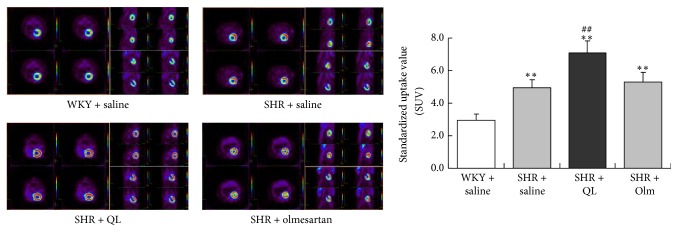
Representative transverse, sagittal, and coronal positron emission tomography (PET) images for myocardial ^18^F-FDG uptake from the four groups of animals were shown. ^18^F-FDG uptake, expressed as standardized uptake value (SUV), was higher in SHRs than in WKY rats. An even higher ^18^F-FDG uptake was noted in SHR + QL group. Olm, olmesartan; ^*∗∗*^*P* < 0.01* versus* WKY + saline group; ^##^*P* < 0.01* versus* SHR + saline group.

Short-axis and longitudinal-axis images of ^18^F-FDG uptake in rat hearts were acquired and provided excellent contrast quality and relatively no liver contamination. SUV was calculated to evaluate ^18^F-FDG uptake by heart. Compared with age-matched WKY rats, PET acquisition showed an increase in myocardial ^18^F-FDG uptake in SHRs at 14 months old, an age when SHRs displayed stable compensated concentric hypertrophy, diastolic dysfunction, and concomitant supernormal systolic function [19]. A rather higher ^18^F-FDG uptake could be observed in SHR + QL group than in SHR + saline group, suggesting that QL improved cardiac diastolic function somewhat associated with enhancement of myocardial glucose uptake. However, olmesartan seemed to have slight influence on ^18^F-FDG uptake with no significance (Figure 4).

### 3.4. QL Promoted Protein Expressions Facilitating Cardiac Energy Metabolism

**Figure 5:**
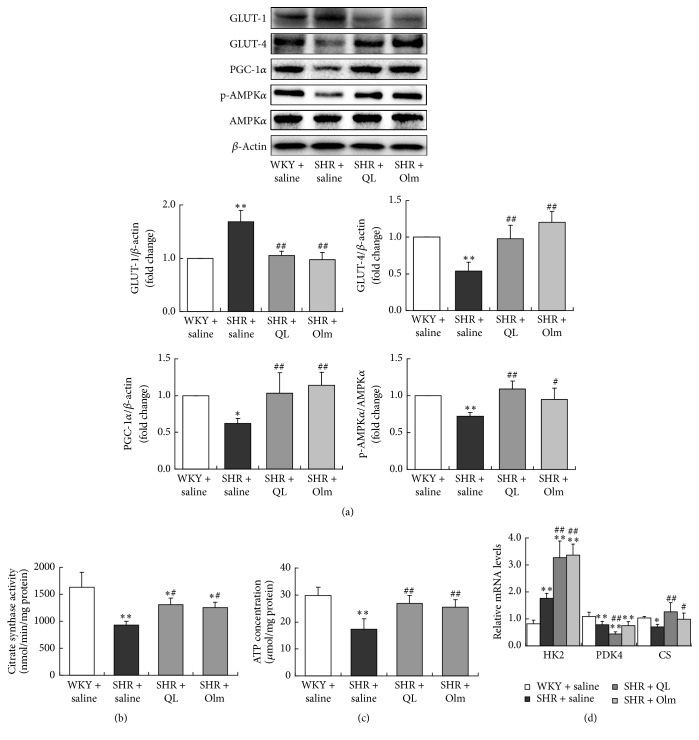
Mitochondrial function, along with relevant protein and gene expressions in the heart of experimental animals. (a) Relative protein expressions including GLUT-1, GLUT-4, PGC-1*α*, and p-AMPK*α* in left ventricular myocardium of the four animal groups. GLUT-1, glucose transporter-1; GLUT-4, glucose transporter-4; PGC-1*α*, peroxisome proliferator-activated receptor coactivator-1*α*; p-AMPK*α*, phospho-AMP-activated protein kinase *α*. (b) Citrate synthase activity measured in heart homogenates and expressed as nmol/min per milligram of total protein. (c) ATP concentration detected in LV myocardial tissues of the four animal groups. (d) Relative HK2, PDK4, and CS mRNA expressions in left ventricular myocardium of the four animal groups. HK2, hexokinase-2; PDK4, pyruvate dehydrogenase kinase-4; CS, citrate synthase. Olm, olmesartan; ^*∗*^*P* < 0.05* versus* WKY + saline group; ^*∗∗*^*P* < 0.01* versus* WKY + saline group; ^#^*P* < 0.05* versus* SHR + saline group; ^##^*P* < 0.01* versus* SHR + saline group.

Several crucial proteins participating in myocardial energy metabolism were analyzed in the present study. In the first place, the protein expressions of GLUT-4, phospho-AMPK*α*^Thr172^, and PGC-1*α* were significantly reduced in SHRs compared with WKY rats, while GLUT-1 expression was elevated. In the next place, QL-treated animals exhibited a significant increase of GLUT-4, phospho-AMPK*α*^Thr172^, and PGC-1*α* expressions in myocardium in comparison to SHR + saline group. Nevertheless, GLUT-1 expression was significantly decreased in SHR + QL group. Olmesartan exerted a similar effect on these proteins as QL (Figure 5(a)).

### 3.5. QL Increased Myocardial CS Activity and ATP Production

CS activity, a classic biomarker of mitochondrial content and density [20], was assessed in our study. A significantly impaired CS activity was observed in the heart of SHRs compared with WKY rats (932.5 ± 69.5 nmol/min/mg* versus *1631.8 ± 275.5 nmol/min/mg, *P* < 0.01). Both QL (1307.7 ± 125.5 nmol/min/mg) and olmesartan (1255.7 ± 95.3 nmol/min/mg) enhanced myocardial CS activity (*P* < 0.05), indicating a higher mitochondrial mass. Concomitantly, SHRs showed a significantly lower ATP content in myocardial tissues than age-matched WKY rats (17.3 ± 4.0 *μ*mol/mg* versus *29.9 ± 3.0 *μ*mol/mg, *P* < 0.01), while QL-treated (26.8 ± 2.9 *μ*mol/mg) and olmesartan-treated (25.5 ± 2.9 *μ*mol/mg) animals revealed a significantly higher ATP production in heart than SHR control (*P* < 0.01) (Figures 5(b) and 5(c)).

### 3.6. QL Regulated Key Enzymes Expression Involved in Glucose Aerobic Oxidation

To further investigate whether QL regulates glucose utilization and oxidative phosphorylation, several genes expressions related to glucose aerobic oxidation were studied. HK2, PDK4, and CS regulate glycolysis, acetyl-CoA generation, and tricarboxylic acid cycle (TCA cycle), respectively. The results of our study showed a significantly higher mRNA level of HK2 and lower levels of PDK4 and CS in SHRs than in WKY rats, indicating an enhancement of glycolysis and acetyl-CoA generation in SHRs. However, TCA cycle, the terminal stage of aerobic oxidation, was hindered in SHRs due to lower expression of CS. QL significantly upregulated HK2 and CS mRNA expressions and downregulated PDK4 expression, promoting the aerobic oxidation in SHRs. Olmesartan could also upregulate mRNA levels of HK2 and CS in SHRs but failed to impact on PDK4 (Figure 5(d)).

## 4. Discussion

The main findings of the present study could be summarized as follows: (1) QL, a traditional Chinese medicine, could improve cardiac diastolic function, alleviate cardiac hypertrophy, fibrotic deposits, and myocyte apoptosis in SHRs. (2) QL enhanced myocardial glucose metabolism and ATP production in SHRs, which might be responsible for its protective effects on diastolic function. (3) Upregulation of AMPK/PGC-1*α* axis and several key enzymes related to glucose aerobic oxidation were involved in such improvement of energy reserves and metabolic state.

SHR is a well-established model of genetic hypertension and has been widely studied to mimic cardiac diastolic dysfunction and HFpEF [15, 16, 21]. The compensated response to pressure overload is LV concentric hypertrophy and diastolic dysfunction, characterized as increased wall thickening and stiffness. In the present study, TDI parameters including *E*′/*A*′ combined with *E*/*E*′ were determined to assess the protective effect of QL on diastolic function, which were less preload dependent and could better represent LV relaxation and compliance [22]. As a result, a definite improvement on LV diastolic function and compliance was observed in SHRs treated with QL independent of systolic pressure. Nevertheless, traditional Doppler parameters such as *E*/*A* showed no significant difference among our animal groups, which was mainly load dependent and could barely reflect diastolic function precisely. Olmesartan could also attenuate cardiac hypertrophy and improve diastolic function, consistent with our previous study [23]. Hypertension could have deleterious effects on heart in the form of hypertrophy, fibrosis, and apoptosis [24], which may account for the impaired cardiac diastolic performance in SHRs. Our histological analysis demonstrated the antihypertrophy, antifibrosis, and antiapoptosis effects of QL in a microscopic scale. TNF-*α* and TGF-*β*1 are well-known cytokines involved in apoptosis and fibrosis. In the present study, the elevated plasma levels of NT-proBNP, TNF-*α*, and TGF-*β*1 in SHRs were reversed by QL and olmesartan, suggesting that QL considerably blocked the progression of heart dysfunction.

Since insulin resistance and glucose metabolism disorders are independently associated with LV diastolic dysfunction [6], we shed great light on cardiac glucose metabolism in our rat model. ^18^F-FDG PET has proved to be a vivid and explicit noninvasive technique for evaluating myocardial glucose uptake with a quantitative SUV calculated. A higher ^18^F-FDG uptake could be seen in SHRs than in WKY rats, while QL further promoted myocardial glucose uptake in SHRs. In 14-month-old SHRs, LV hypertrophy has not yet progressed to congestive heart failure, with stimulated glucose uptake and oxidation as compensation. QL actively accelerated myocardial glucose uptake so as to provide adequate metabolism substrate. In contrast, olmesartan did not enhance glucose uptake, probably due to a lower blood pressure with a lower oxygen requirement.

Quite a lot of factors participate in glucose transportation, glycolysis, and oxidative phosphorylation. As a modulator of energy metabolism in the heart, AMPK is suggested to modulate fatty acid oxidation and glucose utilization. It could also activate PGC-1*α* by phosphorylation, which is a major upstream regulator of lipid catabolism, oxidative metabolism, mitochondrial metabolism, and biogenesis [25]. Mitochondrial complexes deficiency as a result of compromised AMPK/PGC-1*α* signaling has been observed in rodent models of cardiac hypertrophy and failure [26]. AMPK could also stimulate GLUT-4 translocation from an intracellular vesicle to plasma membrane and induce a more efficient storage and utilization of glucose [27]. Unlike GLUT-4, the insulin independent glucose transporter GLUT-1 is a major mediator of basal cardiac glucose uptake, which keeps at low level under physiological conditions and shows compensatory increase under pathological circumstances. In hypertension, there is profound downregulation of GLUT-4 due to cardiac insulin resistance, together with induction of GLUT-1 expression compensating for the loss function of GLUT-4 [28, 29]. In spite of impaired myocardial energetics in cardiac hypertrophy, metabolic adaptations occur with a higher rate of glucose uptake, as is shown in previous studies [19]. However, such kind of passive compensatory action seemed unable to provide sufficient oxygen supply for SHR. By activating AMPK/PGC-1*α* axis and restoring the abnormal GLUT-4/GLUT-1 expression in adult SHRs, QL further enhanced glucose uptake and especially optimized glucose utilization, thus improving cardiac energy metabolism. Previous studies have reported that angiotensin II type 1 receptor (AT1 R) blockade favors glucose metabolism in Ang II- and obesity-induced myocardial hypertrophy [5, 30]. In the present study, olmesartan attenuated glucose metabolism disorders but had minor effect on ^18^F-FDG uptake in SHRs, probably because olmesartan ameliorated pressure overload, which led to less energy requirement. However, QL helped to normalize glucose utilization independent of blood pressure.

CS activity has been validated to be strongly associated with the morphological changes in mitochondrial content and easy to measure. It may contribute to substrates oxidation in mitochondrial respiration [31]. Downregulation of PGC-1*α* results in defective mitochondrial biogenesis and ATP generation in HFpEF model. In the present study, we measured CS activity of myocardial homogenates as indicators of mitochondrial content. By upregulating PGC-1*α* expression, QL preserved mitochondrial content and ATP production, maintaining a balance of substrate supply and utilization.

The hallmark of increased glucose metabolism in the hypertrophied heart from SHRs is decreased mitochondrial oxidation and accelerated glycolysis [32]. Increased glycolysis may provide a source of energy in the failing heart but cannot completely replace the deficit in mitochondrial ATP production [33]. Several crucial enzymes from glycolysis to the TCA cycle are dysregulated when cardiac diastolic dysfunction emerges. As the first step of glycolysis, glucose to glucose-6-phosphate is mainly regulated by HK2. In mitochondria, glucose oxidation rates are regulated by the pyruvate dehydrogenase (PDH) complex, while PDK4 could inactivate PDH complex and inhibit the conversion of pyruvate to acetyl-CoA. In accordance with previous studies, our work revealed increased rates of glucose utilization in hearts of SHR with increased hexokinase and decreased PDK4 expression [27, 34], while such compensatory regulation may not be adequate for SHR heart. Furthermore, mRNA of CS, the enzyme initiating the common pathway of aerobic oxidation, TCA cycle, was downregulated in SHRs. Thus it could be inferred that SHR heart is prone to develop anaerobic metabolism while WKY strain for aerobic metabolism, which may also be related to impaired cardiac diastolic performance in SHRs. QL actively upregulated HK2 and downregulated PDK4 mRNA expressions to a greater degree, contributing to better glucose utilization. More importantly, QL upregulated CS mRNA expression, indicating a catalytic potential for cardiac glucose phosphorylation and subsequent aerobic oxidation for direct energy production, which was blunted in SHRs. Angiotensin II can also adversely affect cardiac energy metabolism manifested as both a deficit in energy production and cardiac energy inefficiency [33]. Olmesartan, a classic AT1 R blockade, elicited a prominent promotion for mitochondrial oxidative phosphorylation in our study. However, it failed to act on PDH complex in our SHR model. Further studies are expected to investigate the underlying mechanism.

## 5. Conclusions

In summary, glucose metabolic impairment proved to be tightly associated with the development of cardiac hypertrophy and diastolic dysfunction in SHRs. QL could improve LV diastolic function and inhibit myocardial hypertrophy, fibrosis, and apoptosis, accompanied by an enhancement of glucose uptake, utilization, and oxidative phosphorylation with increased energy production. Unlike AT1R blockade, the protective effect of QL was independent of blood pressure. Though the precise and target signaling bridging cardiac glucose metabolism and diastolic function were not studied in the present issue, we argued that QL facilitated glucose metabolism in SHR-induced cardiac hypertrophy and could serve as a novel therapy for HFpEF.

## Figures and Tables

**Table 1:** Blood pressure and echocardiographic parameters before and 2 months after intervention.

	WKY + saline	SHR + saline	SHR + QL	SHR + Olm
Systolic blood pressure (mmHg)				
Baseline	126.8 ± 15.1	198.0 ± 25.6^*∗∗*^	201.6 ± 23.7^*∗∗*^	203.6 ± 26.8^*∗∗*^
Treatment	129.6 ± 11.9	196.0 ± 23.3^*∗∗*^	204.0 ± 22.1^*∗∗*^	169.6 ± 13.9^*∗∗*#^
LVEDD (mm)				
Baseline	7.30 ± 0.23	6.86 ± 0.48	6.82 ± 0.50	6.92 ± 0.68
Treatment	7.20 ± 0.27	7.02 ± 0.29	7.06 ± 0.21	6.96 ± 0.34
LVESD (mm)				
Baseline	3.62 ± 0.30	3.08 ± 0.33^*∗*^	2.92 ± 0.23^*∗∗*^	3.06 ± 0.34^*∗*^
Treatment	3.58 ± 0.26	2.90 ± 0.21^*∗∗*^	2.96 ± 0.17^*∗∗*^	2.94 ± 0.11^*∗∗*^
IVS (mm)				
Baseline	1.48 ± 0.19	2.08 ± 0.22^*∗∗*^	2.14 ± 0.18^*∗∗*^	2.18 ± 0.22^*∗∗*^
Treatment	1.42 ± 0.18	2.12 ± 0.26^*∗∗*^	1.76 ± 0.21^*∗*#^	1.66 ± 0.25^##^
LVPW (mm)				
Baseline	2.26 ± 0.29	2.94 ± 0.21^*∗∗*^	3.16 ± 0.30^*∗∗*^	2.96 ± 0.26^*∗∗*^
Treatment	2.22 ± 0.28	3.06 ± 0.13^*∗∗*^	2.76 ± 0.21^*∗∗*#^	2.62 ± 0.19^*∗∗*##^
LVEF (%)				
Baseline	83.06 ± 2.12	87.50 ± 2.44^*∗*^	89.46 ± 3.17^*∗∗*^	88.32 ± 2.63^*∗∗*^
Treatment	83.42 ± 1.80	88.78 ± 1.97^*∗∗*^	88.98 ± 2.65^*∗∗*^	89.48 ± 2.25^*∗∗*^
LVFS (%)				
Baseline	50.32 ± 5.05	55.92 ± 2.86^*∗*^	57.08 ± 3.49^*∗*^	55.66 ± 3.50^*∗*^
Treatment	50.20 ± 4.36	58.70 ± 1.74^*∗∗*^	58.06 ± 2.50^*∗∗*^	57.72 ± 2.18^*∗∗*^
DT (ms)				
Baseline	21.00 ± 1.58	32.20 ± 3.77^*∗∗*^	30.60 ± 6.99^*∗∗*^	31.80 ± 2.59^*∗∗*^
Treatment	20.60 ± 1.67	32.40 ± 2.30^*∗∗*^	25.80 ± 3.96^*∗*##^	25.60 ± 2.88^*∗*##^
*E*/*A*				
Baseline	1.58 ± 0.30	1.57 ± 0.15	1.60 ± 0.21	1.74 ± 0.24
Treatment	1.58 ± 0.20	1.37 ± 0.10	1.46 ± 0.20	1.45 ± 0.24
*E*′/*A*′				
Baseline	1.68 ± 0.23	0.99 ± 0.27^*∗∗*^	0.83 ± 0.30^*∗∗*^	0.88 ± 0.09^*∗∗*^
Treatment	1.67 ± 0.14	0.87 ± 0.17^*∗∗*^	1.21 ± 0.16^*∗∗*##^	1.14 ± 0.14^*∗∗*#^
*E*/*E*′				
Baseline	19.09 ± 2.06	31.20 ± 4.49^*∗∗*^	28.03 ± 2.89^*∗∗*^	29.01 ± 2.98^*∗∗*^
Treatment	19.38 ± 2.75	31.30 ± 4.23^*∗∗*^	23.43 ± 2.23^*∗*##^	23.94 ± 2.02^*∗*##^

Olm, olmesartan; LVEDD, left ventricular end-diastolic diameter; LVESD, left ventricular end-systolic diameter; IVS, interventricular septum thickness; LVPW, left ventricular posterior wall thickness; LVEF, left ventricular ejection fraction; LVFS, left ventricular fractional shortening; DT, deceleration time of *E* wave; *E*/*A*: ratio of peak transmitral velocities of *E* and *A* waves; *E*′/*A*′: ratio of peak tissue velocity of early (*E*′) and late (*A*′) diastolic waves; *E*/*E*′: ratio of *E* to *E*′. Compared with WKY + saline, ^*∗*^*P* < 0.05; ^*∗∗*^*P* < 0.01. Compare with SHR + saline, ^#^*P* < 0.05; ^##^*P* < 0.01.
